# Recurrent implantation failure and sexual function in infertile Iranian women: a comparative cross sectional study

**DOI:** 10.1186/s12978-022-01409-7

**Published:** 2022-04-27

**Authors:** Samira Ghorbani, Parvin Abedi, Khadije Hekmat, Saeed Ghanbari, Narjes Dibavand

**Affiliations:** 1 Midwifery Department, Ahvaz Jundishapur University of Medical Sciences, Ahvaz, Iran; 2 Midwifery Department, Reproductive Health Promotion Research Center, Ahvaz Jundishapur University of Medical Sciences, Ahvaz, Iran; 3 Department of Midwifery, Reproductive Health Promotion Research Center, Ahvaz Jundishapur University of Medical Sciences, Ahvaz, Iran; 4 Department of Biostatistics, School of Health, Ahvaz Jundishapur University of Medical Science, Ahvaz, Iran; 5 Fertility Infertility and Perinatology Research Center, Ahvaz Jundishapur University of Medical Sciences, Golestan BLV, Ahvaz, Iran

**Keywords:** Recurrent implantation failure, Sexual function, Infertility

## Abstract

**Background:**

Recurrent implantation failure (RIF) which means failing to implant after two or more high-quality embryo transfer cycles, affects 3% to 5% of women worldwide. The aim of this study was to assess the relationship between recurrent implantation failure and sexual function in infertile Iranian women.

**Methods:**

This was a comparative cross-sectional study on 180 infertile Iranian women (90 infertile women with recurrent implantation failure and 90 infertile women who did not start infertility treatment). A demographic questionnaire and the Female Sexual Function Index were used for data collection. Data were analyzed using Chi-square, independent t-test, and multiple linear regression.

**Results:**

The mean scores of different domains of sexual function (desire, lubrication, arousal, orgasm, pain, and satisfaction) were significantly lower in the group with RIF compared to the group without RIF. The total score of sexual function was significantly lower in the RIF group compared with the group without RIF (23.11 ± 2.24, vs. 25.99 ± 2.35, p < 0.001). The overall sexual function scores in women with RIF were 2.65 units lower than women without RIF (p < 0.001).

**Conclusion:**

The results of this study showed that women with RIF had significantly lower sexual function than that in women without RIF. Therefore, sexual function issues should be treated as an important component of comprehensive care. This study did not measure the impact of economic factors on sexual function, however, the majority of the sample were classified as having weak or moderate economic status and this, along with the high cost of infertility treatments, could potentially have played a role in the participants' experience. This relationship will need to be investigated in future research.

## Plain Language summary

Recurrent implantation failure (RIF) means inability to implant after two or more high-quality embryo transfer cycles. The aim of this study was to assess the relationship between recurrent implantation failure and sexual function in infertile Iranian women. In this study, 180 infertile Iranian women (including 90 infertile women with recurrent implantation failure and 90 infertile women with no implantation failure) were recruited. A demographic questionnaire and the Female Sexual Function Index were used for data collection. The mean scores of different domains of sexual function (desire, lubrication, arousal, orgasm, pain, and satisfaction) were significantly lower in the group with recurrent implantation failure compared to the group without. The overall sexual function scores in the RIF group were 2.65 units lower than those of women without RIF. Women experiencing recurrent implantation failure may be at a particular risk of reduced sexual function. Therefore, sexual function issues should be treated as an important component of comprehensive care.

## Background

Infertility is defined as failure to achieve pregnancy after 12 months or more of unprotected regular sexual intercourse [1]. In vitro fertilization (IVF) is often the best solution for many couples with different types of infertility [2]. Only one-third of women receiving IVF treatment will achieve an intra-uterine pregnancy [3], and a significant number of patients remain subject to recurrent implantation failure (RIF) [4]. Affecting 3–5% of women worldwide, RIF has also been defined in several studies as the absence of successful implantation following two or more rounds of high-quality embryo transfer, intracytoplasmic injection of sperm, or frozen embryos [5, 6]. The prevalence of RIF in developed countries and developing countries is 3–4%, and 6–7%, respectively, and in Iran, it is about 5–8% [7]. Women with RIF are more likely to experience anxiety (86%), depression (40.8%), stress (24%), and decreased quality of life (70%), which can lead to sexual dysfunction in them [8, 9].

The human sexual response cycle is a physiological process that includes the stages of desire, arousal, orgasm, and suppression. Sexual dysfunction is defined as any problem that occurs in this cycle or failure to reach orgasm [10]. The prevalence of sexual dysfunction in a study in Iran was reported at 31.5% [11]. Women with sexual dysfunction experience mood instability, anxiety, stress, and reduction in life satisfaction [12]. According to previous studies, the rate of anxiety and depression in women with implantation failure is maximized due to uncertainty about the cause of failure, financial problems and the length of re-treatment [13]. There is a significant relationship between the seriousness of depression and anxiety and female sexual satisfaction and performance. This means that with the rise in depression and anxiety, the rate of sexual dysfunction increases and the sexual relationship is done solely for the purpose of having children without any satisfaction in the relationship [14]. In the meantime, following implantation failure, higher stress levels and lower quality of life have been reported, and increased stress and reduced quality of life have been stated to lead to sexual dysfunction and decreased sexual satisfaction [15, 16]. Furthermore, ovarian stimulant drugs used in the infertility treatment process, following implantation failure, will cause hormonal changes and a decrease in hormones that affect sexual function, which may lead to sexual dysfunction in women [17]. Numerous studies have shown that failure to achieve pregnancy may lead infertile women to develop negative beliefs about self, concerns about sexual appeal, feelings of rejection, sexual dysfunction, and marital problems [18, 19].

Infertility itself can reduce sexual function [20], and most studies have focused on one failure in fertility treatment and quality of life, stress, anxiety and depression and sexual function [21]. However, there is paucity of data addressing the relationship between recurrent infertility treatment failure and sexual function. Therefore, this study was designed to assess this relationship between recurrent implantation failure and sexual function among infertile Iranian women.

## Materials and methods

### Study design

This study was a comparative cross-sectional study involving 180 infertile women referring to infertility clinics in Ahvaz, Iran. The study commenced in October and concluded in November 2020. The design of the study was approved by the Ethics Committee of the Ahvaz Jundishapur University of Medical Sciences (Ref ID: IR.AJUMS.REC.1399.073). This study was conducted in two infertility clinics in Ahvaz.

### Participants

In this study, we recruited infertile women with a history of recurrent implantation failure (n = 90) and infertile women that did not start their infertility treatment (n = 90). Inclusion criteria were as follows: age between 18 and 45 years, basic literacy, at least 1-year history of infertility, and history of at least two or more implantation failures. Participants in the control group had a history of infertility but had not yet started treatment. The exclusion criteria were as follows: taking medications that affect sexual function besides of infertility treatment drugs, having psychological disorders (severe depression and anxiety), and diseases that may affect sexual function. Prior to the data collection, written informed consent was obtained from each participant, and anonymity of participants was maintained.

### Sample size

The sample size of this study was calculated following a previous study [22] and based on the following formula:

$$n = \frac{{(Z_{{1 - \frac{\alpha }{2}}} + Z_{\beta } )^{2} (SD_{1}^{2} + SD_{2}^{2} )}}{{(\mu_{1} - \mu_{2} )^{2} }},$$

(µ_1_ = 4.7, µ_2_ = 6, α = 0.05, β = 0.1, SD_1_ = 2.4, SD_2_ = 2.9, Z_β_ = 1.32, Z_1-α/2_ = 1.96). Assuming a 90% power for this study, the final sample size in each group was calculated to be 90 women.

### Measures

To collect data, we used a demographic questionnaire and the Female Sexual Function Index. The demographic questionnaire included questions about age, age of husband, occupation, educational attainment, and economic status. Because the poverty line is not clear in Iran, we just asked participants that how they estimate their economic status. According to the response, if they thought their expenses surpass their income they classified as good, if their income cove all their expenses, they classified as moderate economic status, and if they underestimate their expenses, they classified as poor.

The Female Sexual Function Index contains 19 questions. Two questions are dedicated to sexual desire, four to sexual arousal, four to lubrication, three to orgasm, three to satisfaction, and three to measuring pain. The score for each domain was multiplied by a specific factor: 0.6 for desire, 0.3 for arousal and lubrication, and 0.4 for other domains. The minimum and maximum scores in all areas are 2 and 36, respectively [23]. Scores below 26.5 represent reduced sexual function. The Persian version of this questionnaire is available, and its validity and reliability have been confirmed in other studies [24].

The names of patients who had RIF and those who had infertility but had not yet started treatment were taken from the clinic with the help of a gynecologist. Then, while the women were waiting for the doctor’s visit, they were interviewed and the eligible women were asked if they were willing to participate in the study and then they asked to complete the questionnaires. One of the researchers (SG) was available, in case participants had any questions.

### Statistics

The data were analyzed using SPSS version 22. To check the normality of data, Kolmogorov–Smirnov (K–S) test and Histogram were used.

The independent t-test and Chi-square tests were used for numerical and categorical data, respectively. The multiple Linear regression was used to examine relationship between RIF and sexual function when adjusted for confounding variables. In all cases, we considered the significance level as p < 0.05.

## Results

In this study, 180 infertile women referring to the Ahvaz infertility centers were recruited, of whom 90 had RIF and 90 had no RIF. The sociodemographic information of both groups is shown in Table 1. Except for age (participants in the RIF group were considerably older), women in the two groups did not have any significant difference regarding demographic characteristics. Causes of infertility in the RIF group were female factor (33.3%), male factor (34.4%), and unexplained infertility (32.2%), whereas and in the group without RIF, they included female factor (37.8%), male factor (41.1%), and unexplained infertility (21.1%), (p = 0.239) (Table 2). The histogram with normal curve illustrated that distribution of all data are very close to normal except for those of pain that although histogram was not normal, but according to K–S test it was considered as normal.

**Table 1:** Socio-demographic characteristics of participants in two groups

Variables	With RIF n = 90	Without RIF n = 90	p-value
Mean ± SD			
Age (y)	33.34 ± 5.34	31.03 ± 5.56	0.005
Age of marriage (y)	24.10 ± 6.26	22.76 ± 5.29	0.123
Duration of marriage (y)	9.26 ± 4.45	8.02 ± 4.73	0.071
n (%)			
Occupation of women			0.816
House maker	80 (88.9)	79 (87.8)	
Employee	10 (11.1)	11 (12.2)	
Education of women			0.879
Primary	17 (18.9)	14 (16.7)	
High school	16 (17.8)	14 (15.6)	
Diploma	29 (32.2)	34 (37.8)	
University education	28 (31.1)	27 (30.0)	
Occupation of men			0.947
Unemployed	3 (3.3)	3 (3.3)	
Self-employee	42 (46.7)	41 (45.6)	
Employee	32 (35.6)	35 (38.9)	
Manual worker	13 (14.4)	11 (12.2)	
Education of men			0.690
Primary	12 (13.3)	8 (8.9)	
High school	10 (11.1)	14 (15.6)	
Diploma	33 (36.7)	33 (36.7)	
University education	35 (38.9)	35 (38.9)	
Economic situation			0.056
Poor	20 (22.2)	9 (10.0)	
Moderate	60 (66.7)	65 (72.2)	
Good	10 (11.1)	16 (17.8)

**Table 2:** Infertility characteristics of participants in two groups

Variables	With RIF n = 90	Without RIF n = 90	p-value
Mean ± SD			
Duration of infertility (y)	7.48 ± 4.49	4.81 ± 3.29	< 0.001
n (%)			
Cause of infertility			0.239
Men	31 (34.4)	37 (41.1)	
Women	30 (33.3)	34 (37.8)	
Unknown	29 (32.2)	19 (21.1)	
Type of infertility treatment			0.358
IVF	71 (78.9)	69 (76.7)	
Other methods	19 (21.2)	21 (23.3)	

*IVF* In vitro fertilization

As evident in Table 3, the mean scores of different domains of sexual function, including (desire, lubrication, arousal, orgasm, pain, and satisfaction) was significantly lower in the group with RIF in comparison to the group without RIF. Moreover, the total score of sexual function was significantly lower in the group with RIF compared with the group without RIF (23.11 ± 2.24 vs. 25.99 ± 2.35, p < 0.001). Nearly 90% of infertile women with RIF had sexual dysfunction.

**Table 3:** Comparison of female sexual function index in two groups with and without RIF

Sexual dysfunction	With RIF n = 90	Without RIF n = 90	p-value
Desire	3.43 ± .68	4.07 ± 0.52	< 0.001
Arousal	3.77 ± 0.79	4.34 ± 0.75	< 0.001
Lubrication	3.01 ± 0.55	3.21 ± 0.42	0.006
Orgasm	3.56 ± 0.65	3.91 ± 0.50	< 0.001
Satisfaction	4.60 ± 0.90	4.99 ± 0.62	0.001
Pain	4.74 ± 1.09	5.41 ± 0.64	< 0.001
Total sexual dysfunction	23.11 ± 2.49	25.99 ± 2.32	< 0.001

Table 4 shows the results of linear regression for the relationship between the RIF and the components of sexual function when adjusted for confounding variables (i.e., age and economic status). As evident from this table, compared with women without RIF, sexual desire, sexual arousal, and lubrication in women with RIF were 0.61 (p < 0.001), 0.57 (p < 0.001), and 0.21 (p = 0.007) units lower, respectively. Also, orgasm and sexual satisfaction were 0.33 (p < 0.001), and 0.32 (p < 0.001) units lower in women with RIF, respectively.

**Table 4:** Relationship of recurrent implantation failure (RIF) with sexual function using multiple linear regression

Dependent variable	Dependent variables	Coef. (SE)	t	p-value	95% CI of coef	
					Lower	Upper
Sexual desire	Age	− 0.003 (0.008)	− 0.38	0.707	− 0.02	0.01
	Group (RC: No failure)	− 0.61 (0.09)	− 6.58	< 0.001	− 0.79	− 0.43
	Economic situation (RC: Poor)					
	Moderate	0.17 (0.13)	1.32	0.190	− 0.08	0.41
	Good	0.22 (0.17)	1.35	0.178	− 0.10	0.55
Sexual arousal	Age	− 0.02 (0.01)	− 1.70	0.091	− 0.04	0.002
	Group (RC: No failure)	− 0.57 (0.12)	− 4.82	< 0.001	− 0.80	− 0.33
	Economic situation (RC: Poor)					
	Moderate	− 0.005 (0.16)	− 0.04	0.971	− 0.32	0.31
	Good	0.15 (0.21)	0.72	0.473	− 0.26	0.57
Lubrication	Age	− 0.002 (0.006)	− 0.43	0.670	− 0.02	0.01
	Group (RC: No failure)	− 0.21 (0.08)	− 2.72	0.007	− 0.36	− 0.05
	Economic situation (RC: Poor)					
	Moderate	− 0.12 (0.10)	− 1.16	0.248	− 0.32	0.08
	Good	− 0.04 (0.13)	− 0.31	0.754	− 0.31	0.22
Orgasm	Age	− 0.005 (0.01)	− 0.62	0.538	− 0.02	0.01
	Group (RC: No failure)	− 0.33 (0.09)	− 3.71	< 0.001	− 0.51	− 0.16
	Economic situation (RC: Poor)					
	Moderate	− 0.01 (0.12)	− 0.10	0.918	− 0.26	0.23
	Good	0.01 (0.16)	0.08	0.935	− 0.31	0.33
Sexual satisfaction	Age	− 0.02 (0.01)	− 1.64	0.104	− 0.04	0.003
	Group (RC: No failure)	− 0.32 (0.12)	− 2.72	0.007	− 0.55	-0.09
	Economic situation (RC: Poor)					
	Moderate	0.27 (0.16)	1.69	0.093	− 0.05	0.59
	Good	0.15 (0.21)	0.71	0.481	− 0.27	0.57
Pain	Age	0.001 (0.01)	0.11	0.909	− 0.02	0.02
	Group (RC: No failure)	− 0.61 (0.14)	− 4.53	< 0.001	− 0.88	− 0.35
	Economic situation (RC: Poor)					
	Moderate	0.56 (0.18)	3.04	0.003	0.20	0.93
	Good	0.52 (0.24)	2.15	0.033	0.04	0.99
Total sexual function	Age	− 0.04 (0.03)	− 1.35	0.178	− 0.11	0.021
	Group (RC: No failure)	− 2.65 (0.37)	− 7.17	< 0.001	− 3.39	− 1.93
	Economic situation (RC: Poor)					
	Moderate	0.87 (0.51)	1.70	0.090	− 0.14	1.87
	Good	1.02 (0.66)	1.53	0.127	− 0.29	2.33

*Coef* coefficient, *SE* standard error, *CI* confidence interval, *RC* reference category

Pain score in women with RIF was 0.61 unit higher than that in women without RIF (p < 0.001).

Finally, the overall sexual function score in women with RIF were 2.65 units lower than that in women without RIF (p < 0.001).

## Discussion

The purpose of this study was to compare the sexual function of two groups of infertile women with or without a RIF. The findings showed that women with RIF received a lower score in all areas of sexual function, including sexual desire, arousal, lubrication, orgasm, and satisfaction, and higher scores for pain. Although several studies have assessed the sexual function of infertile women and women under treatment for infertility, this is the first study to address sexual function in women who have RIF. In the following, we review the results of other studies regarding the relationship between infertility treatment and sexual function in women.

Starc et al. [25] conducted a systematic review on infertility and sexual dysfunction and found that infertility negatively affects the sexual function of infertile couples in this way that 43% to 90% of infertile women had sexual dysfunction and lower sexual satisfaction was more dominant. The results of the present study are similar to those of Starc et al. as almost 90% of women in the RIF group had sexual dysfunction. This rate is much higher than that in infertile Iranian women, as according to the results of a systematic review including 18 studies and involving 3419 infertile women, 64.3% of Iranian Infertile women suffered from sexual dysfunction [26]. This higher rate of sexual dysfunction in our study can be related to failure in infertility treatment.

In the present study, the results of multiple linear regression showed that sexual desire in women with RIF was 0.61 unit less than that in women without RIF. Also, sexual arousal, lubrication, orgasm, and sexual satisfaction in the women with RIF were 0.57, 0.21, 0.33, and 0.32 units lower than those in women without RIF, respectively. Finally, women with RIF had more pain and their sexual function was 2.65 units lower than that in women without RIF. We could not find a study compared the sexual function of women with and without RIF. But a study by Bakhtiari et al. [27] that conducted on 236 infertile women who referred for infertility treatment showed that the prevalence of sexual dysfunction was 55.5%, with the more prevalence for dyspareunia (28%). However, sexual function in women with RIF in our study is much higher than the study of Bakhtiari et al. and we can attribute this increase to repeated failures in infertility treatment.

In another study, Leiblum et al. [28] examined sexual function in three groups of successful IVF, unsuccessful IVF, and adoption of a child. They found that women who succeeded in their IVF were more satisfied with their sexual function than women without success and those who adopted a child. Although, in the present study, we recruited two groups of women, one with RIF and one group who were infertile, but did not start their treatment, the results of the reduced sexual function in women who had RIF were comparable and in agreement to the results of the group that had one failure in the treatment in Leiblum et al.’s study.

Evidence also showed that women with RIF experience more anxiety, stress, and depression, and these factors may cause sexual dysfunction [29], but it is not clear that whether or not stress causes infertility [30]. Furthermore, stress, anxiety and depression have been found to have a negative relationship with sexual function in women, with depression exerting the most negative effect [31].

Similar to our investigation, Okobi, in a systematic review including eight studies, found that overall sexual function was impaired more in infertile women, especially in the areas of pain, sexual desire, and lubrication. He concluded that other factors such as psychosocial, cultural, and economic, play an important role in the sexual dysfunction of infertile women, especially in developing countries [32]. In the present study, in addition to psychological factors, financial issues are definitely effective in reducing sexual function in women with RIF, because in Iran many infertility treatments are not covered by insurance. Furthermore, although in the present study, the economic status of women was not significantly different between the two groups, most of the women in the RIF group had weak or moderate economic status.

## Strengths and limitations of the study

To the best of our knowledge, this is the first study to deal with the relationship between RIF and sexual function in infertile women, and the results can promote knowledge in this regard. Despite its strengths, this study has certain limitations. First, we did not recruit women randomly. Second, talking about sexual issues in Iranian culture is a taboo, and the information about sexual function provided by the participants in this study may have been affected by this issue. Furthermore, we did not assess the stress, anxiety, and level of hormones of the participants, and these factors may have affected sexual function in addition to RIF.

And finally, this study did not measure the impact of economic factors on sexual function, however, the majority of the participants were classified as having weak or moderate economic status and this, along with the high cost of infertility treatments, could potentially have played a role in the participants' experience. This relationship will need to be investigated further in future research.

## Conclusion

Women with recurrent implantation failure may be particularly at risk of decreased sexual function. Sexual function problems may have a significant impact on the overall quality of life during infertility treatment. Therefore, sexual function should be assessed during infertility treatment and necessary counseling should be provided for infertile women, especially for those that have recurrent implantation failure. However, the sexual function of partners of infertile women, anxiety, stress and the level of hormones are also important factors that should be assessed in future studies.

## Data Availability

Data used in this study will be available upon reasonable request from the corresponding author.

## Abbreviations

RIF: Recurrent implantation failure
IVF: In vitro fertilization
IUI: Intrauterine insemination
